# Melanoma Diagnosis in the Mihm Era—And Beyond

**DOI:** 10.1111/cup.14742

**Published:** 2024-11-03

**Authors:** David E. Elder

**Affiliations:** ^1^ Hospital of the University of Pennsylvania Philadelphia Pennsylvania USA

**Keywords:** diagnosis, dysplastic nevi, history, melanoma, overdiagnosis, vertical growth phase

## Abstract

During the illustrious career of Martin C. Mihm Jr., MD, the diagnosis of melanoma underwent significant changes, to which he made many contributions. In early descriptions, melanomas were fungating tumor masses that were obviously malignant, and highly lethal. In seminal work by Dr. Mihm and his mentor, Wallace H. Clark, Jr., MD, the early phases of development of these neoplasms were recognized and distinguished from the more advanced disease. It was generally believed that the early stage of melanoma, termed radial growth phase (RGP) and characterized by absence of vertical growth phase (VGP) and by favorable microstaging attributes could be recognized, excised, and cured, thus preventing the development of advanced disease. However, strenuous efforts in this direction over several decades have resulted in little or no change in mortality, leading to the recognition that many of these neoplasms, at least, may not be true biological malignancies, and to the conclusion that overdiagnosis commonly occurs in this disease, which is defined as representing diagnosis as melanoma of a neoplasm that would not have had the capacity to cause death or symptoms in the lifetime of the host. Although there may be other subsets of neoplasms in this category, an important category of overdiagnosis in melanomas is concentrated in T1a melanomas that lack VGP. If these neoplasms can be recognized with sensitive and specific criteria, which may already be available, changes in terminology may be appropriate, recognizing that some of them may have low malignant potential, whereas others may have no capacity at all for metastasis and may not warrant the use of the term “melanoma.”

## 1 Introduction

The career of Dr. Martin C. Mihm Jr., spanned over half a century, during which the diagnosis of melanoma underwent continuous evolution. In Dr. Mihm's first two publications on melanoma in 1969 and 1971, with Clark et al. [1, 2], detailed criteria were enumerated for the diagnosis of melanoma. At that time, melanomas had mostly been regarded as tumorigenic proliferations, in the old sense of “tumor, dolor, rubor, calor,” terms describing the process of inflammation, where the word tumor simply meant a lump or swelling, not a neoplasm as has come to be the current meaning. The two seminal publications emphasized in addition the presence in many melanomas of an adjacent component that was comprised of melanoma cells in the epidermis and, in many instances, in the papillary dermis, which later came to be termed the “radial growth phase” (RGP) of the disease, because it represented a stage where clinically melanomas expanded, as it were, along the radius of an imperfect circle. In contrast, the tumorigenic component of melanomas was termed the vertical growth phase (VGP) [3]. Subsequent studies have documented that the VGP is the source of metastatic disease and, indeed, VGP cells share many properties with the cells of metastases, as will be reviewed in the sections following. In this document, Dr. Mihm's contributions to the evolving classification of melanoma will be reviewed in the context of those of other early seminal contributors, including Wallace H. Clark Jr., Vincent J. McGovern, Richard J. Reed, and A. Bernard Ackerman.

### 1.1 The Classification of Melanoma

In the early publications, the classification of melanoma that is currently used was delineated in the studies of Clark et al. [1]. In the first paper, titled “The Histogenesis and Biologic Behavior of Primary Human Malignant Melanomas of the Skin,” the histogenesis of three forms of malignant melanoma, superficial spreading melanoma (SSM), nodular melanoma (NM), and lentigo maligna melanoma (LMM) was described. Evidence was presented suggesting that SSM and LMM, though evolving at different rates, underwent a long period of superficial growth, followed by the relatively rapid appearance of nodules of deeper invasion within the primary neoplasm. It was speculated that this change in the nature of the primary neoplasm might be due to the appearance of one or more strains of cells of aggressive biologic potential, due perhaps to tumor selection over time. The neoplasms of NM did not have a flat component adjacent to the tumorigenic component as was present in SSM and LMM. It was speculated that this form of melanoma might have developed biological aggression earlier in its stage of evolution, but not that it was a fundamentally different form of the disease. Similar observations were made by Vincent McGovern in Australia, who introduced the term “pagetoid melanoma” for the SSM of Clark and Mihm [4]. The classification was also expanded by the observation by Arrington et al. [5] of another special form of melanoma, acral‐lentiginous melanoma (ALM). These prescient observations have been amply confirmed in more recent studies, which were summarized in the 2018 WHO Classification of Skin Tumors, where nine different “pathways” to melanoma were discussed, in terms of their disparate epidemiology, histogenesis, and genomic characteristics [6, 7]. The neoplasms originally described as SSM, LMM, and NM constitute the most frequent forms of melanoma observed in Western populations. Remarkably, these and also other forms of melanoma, originally classified on morphologic grounds alone, are characterized by distinguishable genomic and epidemiologic characteristics, which separate them from each other, and have implications for the medical management of advanced disease, and for the prevention of melanoma. These characteristics do not clearly separate a distinct category of NM, which therefore, as was predicted by Clark and Mihm, appears to represent an accelerated form of development of one or another of the other subtypes of melanoma. However, NM is clinically and biologically distinct as it presents with different clinical features, namely an often more or less symmetrical nodule that might be quite deep even when not especially broad, often sparsely or not pigmented, compared with a variegated pigmented plaque neoplasm, with or without a nodular component in the skin in the melanomas that have an RGP. NM also accounts for a disproportionate share of the overall deaths from melanoma [8].

### 1.2 Tumor Progression in Melanoma

Extrapolating from these early observations, the notion that melanomas evolve through a process of stepwise tumor progression was developed. Indeed, it was postulated by Clark et al. that “Six evident lesional steps of tumor progression form the neoplastic system that affects the human epidermal melanocyte: (i) the common acquired melanocytic nevus; (ii) a melanocytic nevus with lentiginous melanocytic hyperplasia, that is, aberrant differentiation; (iii) a melanocytic nevus with aberrant differentiation and melanocytic nuclear atypia, that is, melanocytic dysplasia; (iv) the RGP of primary melanoma; (v) the VGP of primary melanoma; and (vi) metastatic melanoma” [9]. Studies were done comparatively of RGP and VGP cells, demonstrating that RGP cells were nontumorigenic in immunodeficient animals, although they could be cultured at least for a time in artificial media. VGP cells, in contrast, and similar in this respect to metastatic melanoma cells, were readily propagated in media and in immunodeficient animals [10] and more recently in xenografts [11]. Studies ex vivo based on careful morphologic and immunohistochemical evaluations in paraffin and frozen sections, demonstrated differences in antigenicity and in proliferative capacity between RGP and VGP, with the latter tending to resemble metastatic melanoma cells [12, 13, 14].

Concurrently, microstaging attributes such as ulceration, Clark's levels of invasion, Breslow's thickness, tumor mitotic rate, and tumor‐infiltrating lymphocytes, were recognized to have predictive value in the determination of prognosis of large groups of melanoma cases, and, less effectively, of individual melanoma cases. This led to the recognition that low stage melanomas, currently recognized as AJCC Stage IA melanomas based on Breslow thickness of 1.0 mm or less and lack of ulceration, have a low rate of metastasis [15]. In a seminal paper by Price et al. [16], histologic criteria for the diagnosis of SSM were presented, formulated on the basis of proven metastatic neoplasms. Of note, several of these criteria (such as single cells, confluence of nests, and pagetoid scatter) related to the in situ and/or RGP component of the neoplasms. Taken together, this evidence for progression from low risk to higher risk stages of melanoma led to the further inference that the diagnosis of melanomas in these early, curable stages, might reduce mortality from the disease, and an emphasis on early detection, facilitated by the propagation of detailed clinical images to facilitate early diagnosis [17], and it was stated that “no one should die of melanoma” [18].

**FIGURE 1:**
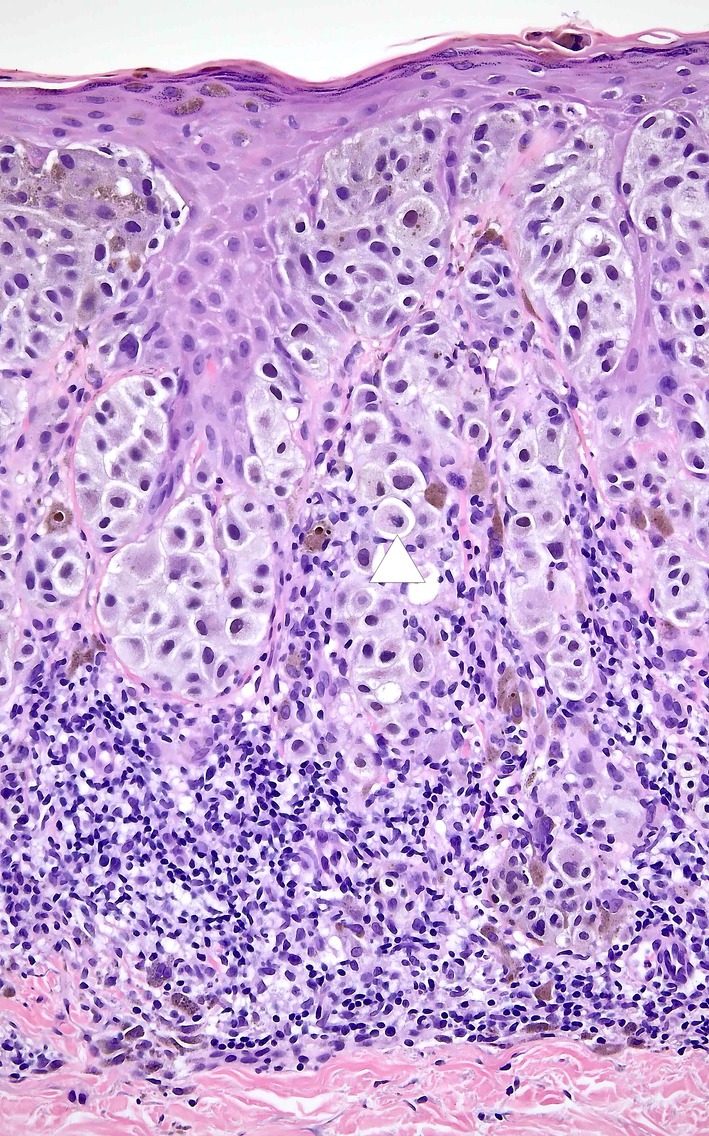
Malignant melanoma, superficial spreading type, with early tumorigenic and mitogenic vertical growth phase. A cellular proliferation of uniformly atypical large epithelioid melanocytes. These are arranged predominantly in nests in the epidermis, with a tendency to confluence of nests and with pagetoid scatter present in the neoplasm though not especially prominent in this field. They have uniformly large somewhat hyperchromatic nuclei. Similar cells protrude into the papillary dermis in relation to a brisk lymphocytic response. The papillary dermis is expanded but not filled, constituting Clark's Level II invasion. The Breslow thickness is 0.4 mm. There are clusters of cells in the dermis that are comparable in size to those in the epidermis and these should be regarded as early tumorigenic VGP in order to maintain the specificity of the “RGP confined” category of melanomas. In addition, there is a single mitosis (white arrow), which is also a defining feature of vertical growth phase.

During this period, clinical follow‐up of what Dr. Mihm referred to as “RGP confined” melanomas demonstrated that these, with exceedingly rare exceptions, were not capable of metastasis [19, 20], and that VGP is associated with potential for metastasizing capability [21]. Studies from the University of Pennsylvania Pigmented Lesion Group documented vanishingly low mortality for patients with melanomas lacking VGP, including the observed 100% 10‐year survival of a group of 161 prospectively diagnosed patients [19, 20]. VGP has been simply defined as the presence of a cluster of cells in the dermis that is larger than the largest cluster in the epidermis, or the presence of any neoplasmal cell mitoses in the dermis [19], whereas RGP‐confined melanoma is defined by the absence of these features (Figure 1). Either of these attributes implies that the neoplasmal cells not only have capacity for survival in the dermis but also for proliferation there, logically representing two important prerequisites for the ability to establish metastatic tumors elsewhere in the body. The diagnosis of VGP in thin tumors has been shown to be reproducible [22]. When analyzed in a large group of melanoma cases, VGP is not always found to be an independent predictor of survival, likely because it is ubiquitously present in the vast majority of melanomas thicker than 0.76 mm, however in one study of 4236 cases, the independent determinants of increased risk of melanoma‐specific death in Stage I and II melanomas were increasing T‐stage, presence of ulceration, presence of mitoses, and VGP [23].

In a recent study from the M‐PATH group, a subset of almost 1000 patients with T1 melanoma, Clark's Level II, age < 44 years, had a 100% prospective observed 7‐year melanoma‐specific survival in a large population‐based database [24]. It is likely that Clark's Level II (defined as melanoma in the dermis that expands but does not fill the papillary dermis) is acting in this study as a surrogate for the absence of VGP. However, a small subset of Clark's Level II neoplasms may qualify as VGP, being tumorigenic without filling an expanded papillary dermis, so that this is not a perfect surrogate for VGP. This study provides direct evidence for the phenomenon of overdiagnosis in a significant subset of T1 melanomas. Of course, overdiagnosis is not limited to T1 melanomas, with a number of other entities being possibly subject to over interpretation, to be discussed in the next section.

### 1.3 Overdiagnosis of Melanoma

Over the last 50 years, the incidence of melanoma has risen rapidly, at one time being considered to represent an alarming epidemic. However, during this time, the rate of mortality from melanoma has changed only little, and in recent years has declined, likely due to the recent development of effective therapy for advanced disease [25] (Figure 1). This discrepancy has led to the consideration that a large number of melanoma diagnoses represent the phenomenon of “overdiagnosis” [26]. According to Chat GTP: “Overdiagnosis is the diagnosis of a medical condition that would never have caused any symptoms or problems. This kind of diagnosis can be harmful if it leads to psychological stress and unnecessary treatments. Broadly, overdiagnosis means making people patients unnecessarily, by identifying problems that were never going to cause harm or by medicalizing ordinary life experiences through expanded definitions of diseases. Overdiagnosis has two major causes: *overdetection* and *overdefinition* of disease” [27].

**FIGURE 2:**
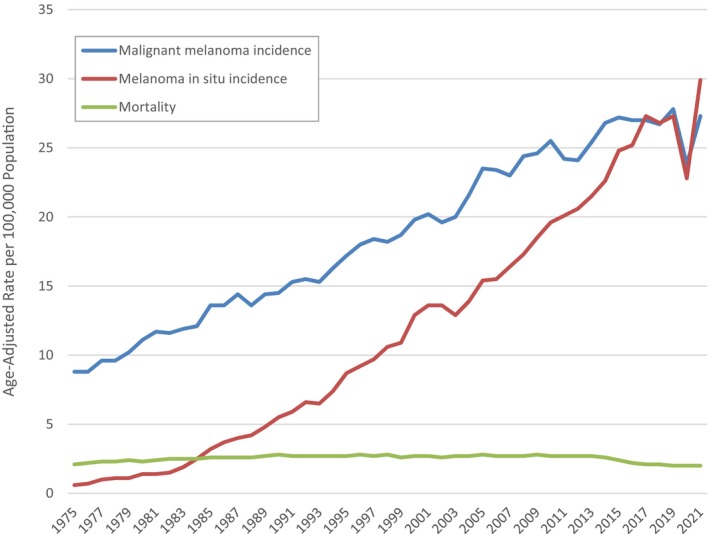
Age‐adjusted incidence rate of invasive and in situ melanoma and mortality rate, 1975–2021. There has been a dramatic increase in incidence, which is even more pronounced for in situ melanoma, without a commensurate increase in mortality. The rate of increase has leveled off in the last 5 years of the data, perhaps due to primary prevention and/or changes in criteria. The fall in incidence in 2020 is likely related to disruption of medical services during the Covid pandemic. The drop in the rate of mortality over the last 5 years began before this and is likely attributable to improved therapy for advanced disease. The incidence of in situ melanoma has increased since the epidemic, and now exceeds that of invasive melanoma. Data were extracted using Surveillance Research Program, National Cancer Institute SEER*Stat software (www.seer.cancer.gov/seerstat) version 8.4.3.

It has been said that the conditions for overdiagnosis of melanoma due to *overdetection* are represented by the presence of indolent lesions in the community, and efforts at early diagnosis, namely screening of asymptomatic individuals or “worried well,” resulting in (over) detection of lesions that would not have caused death or symptoms in the lifetime of the host [26, 28] (Figure 2). Some of these may be indolent neoplasms that would progress only slowly, whereas others, likely the majority, may have no capacity for progression whatsoever.

It is possible to consider other reasons for this phenomenon. For example, early interception of authentic melanomas represents the goal of screening programs, and no doubt this must occur in some instances. However, if these represent a major component of the cases on the “incidence curve” in Figure 1, logic would suggest that there must be other similar neoplasms in the community, not yet diagnosed, that would progress, cause symptoms and ultimately drag up the mortality curve (unless the screening programs have perfectly captured every single example or at least the vast majority of such neoplasms that exist, which of course is an impossibility). Novel therapies that have become available for advanced disease likely explain the falling of mortality beginning in about 2013; however, the magnitude of incident compared with lethal melanomas is not consistent with this trend (e.g., these therapies are only applied to those patients who have disease beyond a certain threshold, which represent a minority of the total incidence of the disease, which is concentrated in Stage I), and in any case, the trend began long before these therapeutic innovations. Evolution of record‐keeping has also been considered as a possibility, with changes in definition of melanoma representing an example of this possibility; however, these changes have been more likely to accentuate than to diminish overdiagnosis as discussed in the next paragraph.

Evidence for *overdefinition* of melanoma was provided by a study at Massachusetts General Hospital that included Dr. Mihm as an author. Neoplasms that had been diagnosed 20 years earlier were re‐evaluated without knowledge of the prior diagnosis and approximately 1/3 of neoplasms that had previously been called nevi, albeit atypical or dysplastic, were classified as melanomas in the later reading [29]. Additional evidence has been obtained by the M‐PATH group, in which it was found that surgical pathologists and dermatopathologists differ in their interpretations of melanocytic neoplasms, limited to the differential diagnosis between severely dysplastic nevi and T1a non‐mitogenic melanomas. Dermatopathologists were more likely to interpret these neoplasms as thin melanomas, compared with surgical pathologists who were more likely to interpret them as nevi, suggesting that heightened sensitivity to the diagnosis of early melanoma may contribute to the overdiagnosis phenomenon [30]. In addition, the M‐PATH studies have documented the existence of considerable uncertainty in this particular field, where the agreement rate among participants in a reproducibility study was literally 50% for this group of cases (severe dysplasia vs. T1a melanoma) [31]. Agreement was much better for the more obviously “wholly benign” or “fully malignant” groups of cases in the study. Molecular and immunohistochemical markers may provide support for classifying neoplasms in this “gray zone” category [32, 33], however these, with some exceptions [34], have in general been evaluated against expert diagnosis, not against mortality, and therefore their biological meaning can be called into question, especially in early disease. Categories of neoplasms that may contribute to overdiagnosis, beyond the scope of this review for more detailed discussion, are listed in Table 1, along with some suggestions for remediation.

**TABLE 1:** Categories of neoplasms that may contribute to overdiagnosis of melanoma.

Categories	Potential remediation
Dysplastic nevi	Rigorous and enhanced criteria
Neoplasms that meet current criteria for MIS	Changes in nomenclature
Nontumorigenic nonmitogenic T1a melanomas	Changes in nomenclature
Spitz tumors that mimic melanoma	Enhanced availability of genomic testing
Other undescribed or poorly described entities	Continuing research

### 1.4 Minimal Risk Melanoma, Zero Risk Melanoma, and Reclassification of Melanoma

**FIGURE 3:**
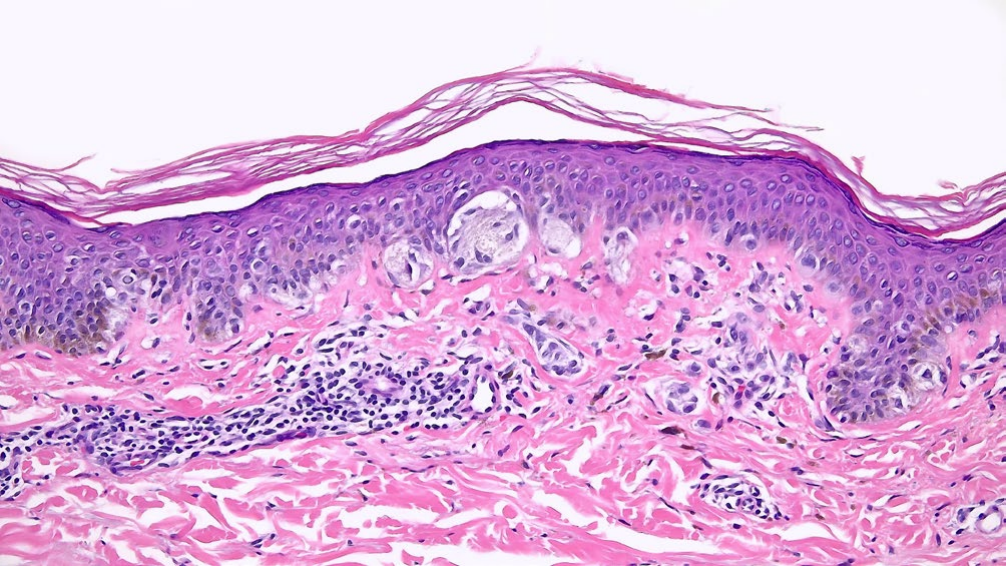
Melanocytic neoplasm of low malignant potential. An atypical melanocytic proliferation, clinically a broad irregular and variegated neoplasm 8 × 7 mm and histologically characterized by large epithelioid cells with moderate to severe nuclear enlargement, and slight irregularity and hyperchromatism, with pagetoid scatter of single cells into the epidermis (more prominent in other nearby fields). In the dermis, there are scattered clusters of cells not larger than those in the epidermis. If this neoplasm is interpreted as melanoma, it can be classified as confined to the radial growth phase (RGP), nonulcerated, Clark's Level II, Breslow thickness 0.3 mm, with an excellent prognosis. Alternatively, it could be classified as severe junctional and dermal melanocytic dysplasia. In either event, it would be judicious to ensure that such a neoplasm is completely excised. Disagreement and uncertainty regarding the diagnosis are concentrated in neoplasms in this general category.

The considerations reviewed above suggest that laudable efforts at early diagnosis and not missing a potentially dangerous precursor or early neoplasm have resulted in a substantial increase in diagnosis as melanoma of cases that would never have progressed to cause injury or symptoms in affected individuals, representing overdiagnosis. Although not in itself life‐threatening, this can have serious consequences for patients in terms of health care costs [35], and also in terms of their life experience, including significant psychosocial consequences [36, 37], and potential insurability [38]. In a recent study, a majority of survivors of localized melanoma, even those who had Stage 0, had high rates of fear of cancer recurrence and intense survivorship experiences that affected their psychological well‐being [39]. Consequences for the healthcare system can be said to include wasted effort in diagnosing these innocuous lesions, and for the research establishment, dilution of the effort required to understand the nature of potentially lethal melanomas, and their management. In consideration of these concerns, it was at one time considered that the term “atypical melanocytic hyperplasia” should be used in preference to “melanoma in situ,” partly for insurance reasons; however, this was resisted in favor of the concept of earlier diagnosis of melanoma [38]. Considerations reviewed above suggest that neoplasms currently termed melanoma that lack VGP may be biologically benign (with exceedingly rare exceptions being a possibility), especially in young individuals, however in the present state of knowledge, we have considered the use of a provisional term “melanocytic neoplasm of low malignant potential” (MNLMP) for these neoplasms (Table 2 and Figure 2) [40]. Reasons for the very rare demonstration of metastasis in association with neoplasms that appear to have lacked VGP could include sampling error in the biopsy specimens, and database errors in the assignment of causes of death. We would emphasize the need for well conducted and well‐funded studies directed at identifying subsets of lesions currently called melanomas for which the predicted probability of survival is 100%. On specific identification of such “zero‐risk” lesions, terminology for these lesions in our opinion should be changed, for example, to severely dysplastic nevus or atypical nevus. For lesions that are to be diagnosed as melanomas, avoiding “zero risk melanoma” overdiagnoses will require incorporating the absence of VGP into the diagnostic algorithm, and these “zero risk” lesions may need to be reclassified as nevi. Specific and sensitive identification of these lesions should enable more focused attention onto the potentially lethal melanomas that are responsible for the substantial mortality that continues to be observed in patients with this disease. These ideas are not new under the sun. The late Wallace H. Clark Jr., a mentor of Dr. Mihm (and of the present author), stated in 1990 “The word melanoma can impact devastatingly on a patient and forever change the nature of his/her life. Patients from all walks of life, including physicians, are greatly disturbed by the diagnosis. It should be rendered only with rigorous evidence. Such evidence is not presently available for neoplasms where the abnormal cells are entirely above the dermal‐epidermal basement membrane zone.” [41] (Figure 3).

**TABLE 2:** Provisional definition of melanocytic neoplasms of low malignant potential (MNLMP).

Based on data from [24]
Age ≤ 70 (less stringent, ≤ 43 would be more stringent, yet perhaps suboptimally sensitive)
Thickness ≤ 0.8 mm
No mitoses
No ulceration
No tumorigenic VGP
Clark Level I or II
No radial growth phase regression
Clinical absence of dynamic changes (indicative of active clinical evolution, e.g., “changing mole”)

*Note:* Some of these criteria may be redundant yet are included for greater stringency and to avoid underdiagnosis. Mortality in cases so defined is vanishingly rare. Continuing research to refine these criteria is needed.

## Conflicts of Interest

The author declares no conflicts of interest.

## Data Availability

Data sharing is not applicable to this article as no new data were created or analyzed in this study.
